# Supplementary material for: The Path of a Cardiac Patient—From the First Symptoms to Diagnosis to Treatment: Experiences from the Tertiary Care Center in Poland
Source: J Clin Med. 2022 Sep 7;11(18):5276. doi: 10.3390/jcm11185276 (PMC9503479; doi:10.3390/jcm11185276)
Supplement: Supplementary file 1 [file jcm-11-05276-s001.zip › jcm-1873522-supplementary.pdf]

# Supplementary material S1- Questionnaire form

## Section 1.- demographic characteristic

**What is your gender?**

_____ (female / male / other- *participants could write their own answers*)

**What is your age (in years)?**

_____ (<20 / 20-30 / 30-40 / 40-50 / 50-60 / >60)

## Section 2.- past medical history

**What cardiac disease have you been diagnosed with?**

***_____** (hypertension / coronary artery disease / chronic heart failure / hypercholesterolemia / arrhythmia / atherosclerosis / other- *participants could write their own answers*)

**Do you suffer from any comorbidities?**

_____ (no / depression and anxiety states / lipid disorders / type II diabetes / hypothyroidism / obesity / chronic pulmonary disease / anemia / stroke / neurologic disorders / other- *participants could write their own answers*)

## Section 3.- the diagnostic process

**How did you know about your cardiac disease?**

_____

**What symptoms caused you anxiety, made you start looking for information about what could be ailing you?**

_____

**Have you been looking for information about the signs, and symptoms of the disease on the Internet?**

_____ (yes / no)

**Where did you look for this information**

_____ (Facebook / Instagram / Medonet / Poradnik Zdrowie / Medycyna Praktyczna/ ABC Zdrowie / Internet groups / First searching results in Google / other- *participants could write their own answers*)

**What was your first step when you suspect it might be a cardiac disease?**

_____ (inpatient visit to an Internal Medicine Doctor or General Practitioner [in the public health sector- free of charge] / inpatient visit to an Internal Medicine Doctor or

General Practitioner [in the commercial sector- paid] / in-patient visit immediately with a specialists / medical teleconsultation [in the public health sector- free of charge] / medical teleconsultation [in the commercial sector- paid] / other- *participants could write their own answers*)

**During the diagnostic process and your searching for information about the disease, have you ever used self-diagnosis tools, e.g. online tests or online surveys?**

_____ (yes / no)

**If yes, which tools have you used?**

_____

**Did you do any additional tests during the diagnosis (blood tests, radiological imaging, etc.)?**

_____ (yes / no)

**How long did the diagnosis process take (i.e. the time from the first symptoms to the moment of the definitive diagnosis) for your disease?**

_____

## Section 4.- current medical conditions and therapeutic process

**Are you taking any medications? If yes, for how long?**

_____

**Do you have any problems with the price or availability of your medications?**

_____ (yes- their price is too high / yes- their availability is limited / no / other- *participants could write their own answers*)

**Where do you most often buy drugs for your disease?**

_____ (at the stationary pharmacy / on the Internet / other- *participants could write their own answers*)

**Have you had any unsuccessful therapies? Were any medications not helping you?**

_____

**Which element of your path from symptoms, by diagnosis, to treatment was/is the most frustrating or caused the most negative emotions for you?**

_____ (searching for symptoms on the Internet / diagnostic tests / visit with the General Practitioner / visit with the Specialist / looking for or buying drugs / other- *participants could write their own answers*)

**Do you have an attending physician?**

_____ (yes / no)

**How often do you have the follow-up?**

_____ (every month / every quarter / twice per year / once per year / less then once per year)

**Have you used the form of teleconsultation/ video consultation with a cardiologist?**

_____ (yes / no)

**Is there any reason stopping you from using the form of remote medical visit?**

_____

## Section 5.- current medical conditions and therapeutic process

**Do you “feel cared” and do you know how to cope with the disease? If not, why?**

_____

**Are you a member of any community, e.g. Facebook groups, internet forums, etc., where you talk about your illness with other patients?**

_____ (yes / no)

**Does the disease cause any restrictions to your current life? Has it changed your previous functioning (e.g. work, hobby, study)? If yes, how? Please share your observations.**

_____ (open question- *participants could write their own answers*)

**If you have additional thoughts about the disease that you want to share - feel free to write below.**

_____ (open question- *participants could write their own answers*)

# Supplementary material S2- Answers in open questions

**Answers in question 26 [section 5]:**

*“Yes - enormous stress due to the unpredictability of the symptoms.”*

*“The disease affected my daily life. It happens that it significantly hinders everyday functioning.”*

*“Yes, it limited me in my hobbies (I actively exercised at the gym), at work (I am less active, I get tired faster and I am less effective).”*

*“The disease limited my options to a great extent. I am bothered by my fatigue.”*

*“I don't think so. I don't feel any discomfort.”*

*“I get tired very quickly. Short walking and stretches became a problem for me.”*

*“I work less.”*

*“Less exercise.”*

*“Yes, to a great extent. I find it difficult to take medications regularly. I avoid efforts. I am concerned about having atrial fibrillation.”*

*“Movement limitations and fatigue disturb me a lot.”*

*“I do not live as if I was healthy”*

*“The disease influenced my life a lot, I had to give up sports activities. I lost my job and went on a disability pension.”*

*“Yes, I had to slow down the pace of my life, I can't drive a car, I can't do what I like (go mushroom picking), limited living comfort, problems with going to the store.”*

*“Partially – physical activity, and movement restriction.”*

*“Reduction of effort.”*

*“Yes, to a large extent.”*

*“I had to quit my job.”*

*“Yes, there are limitations in all areas.”*

*“I don't think so.”*

*“I am impatient, I would like to regain fitness and control as soon as possible.”*

*“Currently, I do not experience any negative effects other than fatigue.”*

*“Walking quickly is difficult for me.”*

*“The too early stage of the disease for me to judge.”*

*“Movement restrictions.”*

*“I don't drink, I don't smoke, I eat less fat. I stopped working because I have manual work.”*

*“Yes, in terms of work.”*

*“Less travel, less alcohol, more sadness.”*

*“I'm retired.”*

*“Fast fatigue, cannot walk and run fast.”*

*“Yes, physical effort limitation.”*

*“To a small extent.”*

*“Limiting physical exertion and slowing down at work.”*

*“In almost every area of everyday life.”*

*“The slowdown in lifestyle, cessation of professional work.”*

*“Restriction of physical activity.”*

*“Yes, to a large extent.”*

*“Lack of possibility of physical activity, difficulty in communicating freely due to shallow breathing.”*

*“Weaker physical condition.”*

*“Lack of mobility.”*

*“I do not lift, do not exercise too much.”*

*“Sometimes I have to give up on leaving the house.”*

*“Lack of physical activity.”*

*“Yes, I'm waiting for heart valve surgery.”*

*“Restriction of physical activity.”*

*“A relaxed lifestyle with adequate physical movement (i.e. walks).”*

*“Less work and effort.”*

*“The disease excluded a normal lifestyle for a while.”*

**Answers in question 27 [section 5]:**

*“It is a disease that can be lived with, but severely limits functioning in society. You have to change your habits overnight. Thank you.”*

*“I learn how to very calm down my body, rest, exercise, and peaceful life. Now, I recommend taking ‘triticco’ [Trazodone] and sport.”*

*“Poor patient care after a heart attack, you have to go privately.”*

*“I need a summary interview with the doctor at the end of my hospitalization.”*

*“Thank you for saving our lives!”*

*“Too long waiting on a helpline and call with the doctor, etc. It is a problem.”*

*“Low availability of medical services.”*

*“Very long process of patient preparation.”*

*“Thank you [doctors] for your work.”*
